# Characterizing the pangenome-based virulome and resistome of uropathogenic *Escherichia coli* from Ecuador

**DOI:** 10.1080/21505594.2026.2707776

**Published:** 2026-08-10

**Authors:** Isaías Mejía-Limones, Ricardo David Avellán-Llaguno, Gabriel Morey-León G, Derly Andrade-Molina

**Affiliations:** a Laboratorio de Ciencias Ómicas, Facultad de Ciencias de La Salud, Universidad Espíritu Santo, Samborondón, Ecuador; b Facultad de Ciencias de la Salud y Desarrollo Humani, Universidad Ecotec, Ecuador

**Keywords:** Virulome, resistome, Uropathogenic, E. coli, pangenome analysis

## Abstract

Uropathogenic *Escherichia coli* (UPEC) is one of the leading causes of bacterial Urinary Tract Infections (UTIs) worldwide. In this study, we characterized the UPEC pangenome in Ecuador, focusing on the virulome and resistome of 142 genomes sequenced using whole-genome sequencing (WGS). Our analysis revealed a structure of 16,732 genes, including a conserved core of 3,385 genes and a dynamic accessory genome of 13,347 genes. The ST131 lineage was predominant, accounting for 42.3 % of the total, whereas the O25:H4 serotype was present in 42.96 % of the cases. The resistome was associated with a large accessory gene repertoire, with 3,712 significant associations identified across 29 antimicrobial resistance genes (ARGs). The virulome was organized into 23 main profiles, with adhesion and siderophore genes being the most abundant. These findings demonstrate the presence of high-risk variants in terms of virulence and resistance circulating in Ecuador, which requires the establishment of improved health control strategies in the region.

## Introduction

Uropathogenic *Escherichia coli* (UPEC) is one of the main causative agents of urinary tract infections (UTIs) worldwide, placing constant pressure on the healthcare systems of countries, particularly those in developing countries [1,2]. It is postulated that the high prevalence of UPEC is due to the vast repertoire of Virulence Factors (VFs) that this group possesses, providing it with advantages in terms of colonization, invasion, and persistence in the urinary tract, in addition to the increasing antimicrobial resistance (AMR) that it possesses, which compromises established therapeutic strategies [3]. Among UPEC strains isolated from Ecuador, the predominant virulence determinants identified are *fimH*, *iutA*, and *sat* genes [4–6], whereas other VFs, such as *cnf1* and *hlyA*, are less prevalent but play a significant role in inducing tissue damage and facilitating immune evasion. Furthermore, the *papC* gene, which encodes P fimbriae, is crucial for adherence to distinct UPEC populations. The combined presence of these VFs enables UPEC strains to efficiently colonize the urinary tract, evade host defenses, acquire essential nutrients, and persist despite antibiotic pressure [7–10], exacerbating the severity and recurrence of UTIs in Ecuador.

Recent advances in whole-genome sequencing and pangenome analysis have revolutionized our ability to delve into the comprehensive genetic landscape of UPEC populations, enabling the characterization of their core and accessory genomes, including VFs and Antibiotic Resistance Genes (ARGs), encompassing acquired and intrinsic resistance determinants [11–13]. Pangenome studies of UPEC have revealed extensive genomic plasticity, with a conserved core genome supplemented by a dynamic accessory genome enriched in genes encoding adhesins, toxins, iron acquisition systems, and diverse antimicrobial resistance elements, such as β-lactamases and efflux pumps [14–16]. These genomic insights are critical for understanding the molecular basis of UPEC pathogenicity and resistance and their epidemiological dissemination, including the prevalence of high-risk clones such as *ST131* and *ST1193*, which have been documented globally and in Latin America [17–19]. However, there remains a paucity of data on the pangenome-related virulome and resistome of UPEC strains circulating in Ecuador, a region with unique demographic and antibiotic usage profiles that may influence the local genomic architecture of these pathogens.

This study aimed to fill this knowledge gap by performing a comprehensive genomic analysis of UPEC isolates from Ecuador, focusing on their pan-genomic composition and associated virulence factor (VF) and ARG profiles. Using this approach, we sought to elucidate the genetic determinants underlying UPEC pathogenicity and multidrug resistance in this understudied population. Such insights are essential for informing targeted therapeutic strategies, guiding empirical treatment, and implementing effective infection control measures tailored to the Ecuadorian context of this study.

## Materials and methods

### Data collection, quality assessment of reads, assembly, and typing process from whole genome sequencing strains from Ecuador

Systematic screening of NCBI BioProjects was performed to select datasets meeting specific inclusion criteria: availability of raw reads for uniform assembly and origin from urinary tract samples. Two BioProjects, PRJNA1168579 and PRJNA1293794, were identified, yielding a total of 145 *E. coli* isolates. Notably, these accessions did not include the metadata necessary for phenotypic-genotypic correlations or clinical details regarding the host patients (Supplementary Table 1). The integrity of the reads was assessed by FastQC [20], and reads exhibiting a Q-score of 30 or lower were subjected to trimming via Trimmomatic v0.39 [21]. The SRA Toolkit v3.2.1 (https://github.com/ncbi/sra-tools) was used to download reads, and MEGAHIT v1.2.9 [22] was used to assemble the 145 *E. coli* genomes by de novo assembly, with a minimum contig size of 200 bp. To improve the quality of the assembly, RagTag v2.1.0 v2.1.0 [23] perform scaffolding, using the *Escherichia coli* O25b:H4-ST131 genome (Accession number: GCF_000285655.3) as a reference; this reference genome was chosen due to its similarity to most of the genomes in the study. To assess the quality of the genomes, CheckM v1.2.5 [24] determines completeness and contamination and QUAST v5.3.0 (https://github.com/ablab/quast). The criteria used to select genomes for further analysis were those with a completeness score of ≥95% and contamination < 5%. Under these parameters, three genomes failed QC and were excluded, leaving 142 genomes for the analysis. For the subsequent typing of the assemblies, MultiLocus Sequence Typing (MLST) v2.23.0 [25] determines the sequence type (ST). Simultaneously, ECTyper v2.0.0 [26], utilizing the v1.0.0 database, was employed to ascertain the pathotype and serotype. Notably, ECTyper is restricted to identifying the seven diarrheogenic pathotypes: DAEC, EAEC, EHEC, EIEC, EPEC, ETEC, and STEC.

### Annotation, pangenome, virulome, and resistome analysis

Genome annotation was performed using Bakta v1.12.0 with the full database [27], which produced GFF files that were used as input files for the pangenome analysis. The pangenome was constructed using PPanGGOLiN v2.3.0 [28] to infer and visualize genomic partitions, including persistent (present in almost all genomes), shell (present at intermediate frequencies within the species), and cloud gene families (present at low frequency within the species). The complete PPanGGOLiN workflow (*all*) was executed to generate graphical representations of pangenome structure and gene family distribution across the analyzed *Escherichia coli* genomes. According to Gautreau et al., the PPanGGOLiN graphical model ensures that the structure of the pangenome remains robust in the context of fragmented assemblies. Additionally, PanGP [29] was used to explore gene distribution profiles and pangenome evolution graphically. The persistent gene alignment was generated using MAFFT v7.526 [30]. A maximum-likelihood phylogeny based on the persistent genome was reconstructed using IQ-TREE v2.4.0 [31] under the TIM2+I+G substitution model, with branch support estimated from 1,000 ultrafast bootstrap replicates.

ARGs and VFs were identified using ABRicate v1.4.0 [32] against the NCBI AMR database (May 2026) [33] and the Virulence Factor Database (VFDB) (May 2026) [34], respectively. Resistance and virulence genes were considered present when sequence alignments met thresholds of ≥80% coverage and ≥80% nucleotide identity, criteria selected to prioritize high-confidence and predominantly complete gene matches. Under these parameters, detected resistance gene variants, including *bla* gene families, were retained when multiple alleles met the identity and coverage thresholds and were treated as distinct gene variants in the presence/absence matrix, whereas partial or fragmented hits below the coverage threshold were excluded to avoid incomplete gene calls. These outputs were integrated and mapped onto the phylogenomic tree using iTOL (https://itol.embl.de/) to visualize the resistome, virulome, and genomic patterns distributions.

Pyseer v1.4.0 [35] was used to investigate associations between antimicrobial resistance genes (ARGs) and accessory gene content within the *Escherichia coli* pangenome. Presence/absence matrices derived from ABRicate outputs, and PPanGGOLiN gene family partitions were analyzed using a linear mixed model (LMM). To account for population structure and genetic relatedness among isolates, a kinship matrix derived from the persistent-genome phylogeny was incorporated into the model. In addition, sequence type (ST) was included as a fixed-effect covariate to minimize potential bias associated with clonal expansion, particularly the predominance of ST131 isolates within the dataset.

Accessory genes and ARGs with a minor allele frequency (MAF) below 5% were excluded prior to analysis. Statistical significance was assessed using likelihood ratio test *p*-values generated by Pyseer, and multiple-testing correction was subsequently performed using the Benjamini – Hochberg (BH) procedure. Associations with an adjusted *p*-value < 0.05 were considered statistically significant.

Significant ARG – accessory gene associations identified by Pyseer were further curated to facilitate biological interpretation. Associations involving hypothetical proteins, uncharacterized proteins, proteins of unknown function were excluded from downstream analyses. The remaining genes were classified according to their predicted biological functions, including antibiotic resistance, cell wall and capsule, DNA metabolism, metabolism, metal resistance, mobile elements and virulence (Supplementary Table 2).

Finally, to determine the presence of virulence gene operons, an operon was considered present when at least 60% of its constituent genes were detected within the same contig in the pre-scaffolding assemblies. To identify virulence-associated profiles, a binary presence/absence matrix of virulence operons was generated for all isolates. Each unique combination of operon presence and absence was considered a distinct virulence profile. Isolates exhibiting identical binary profiles were assigned to the same profile for downstream comparative analyses [36].

## Results

### Characteristics of the assembly, annotation, and typification

The assembly of UPEC genomes yielded an average N50 of 4,887,930 bp, a mean genome size of 5.33 Mb, an average GC content of 50.71 %, and a mean of 386 contigs per genome. Genome quality assessment indicated high overall completeness, with an average completeness value of approximately 99.89%, while estimated contamination remained low across the dataset. (Table 1) Functional annotation with Bakta identified an average of 4,954 coding sequences per isolate. MLST revealed ST131 as the most prevalent sequence type (42.25 %, 60/142), followed by ST38 (6.34 %) and ST44 (4.93 %). Genome-wide serotyping revealed a high prevalence of serotype O25:H4 (42.96 %, 61/142), which is commonly associated with ST131. Other detected serotypes included O-negative:H10 (-:H10, 4.93 %), O-negative:H4 (-:H4, 4.93 %), and O75:H5 (4.23 %), highlighting the diversity among circulating *E*. *coli* strains (Supplementary Table 3).

**Table 1.** Genomic features and assembly quality assessment of the *Escherichia coli* isolate collection. A comprehensive summary of the genomic attributes of the 145 *E. coli* isolates included in the investigation, detailing the distributions of sequence types and serotypes, as well as indicators of assembly quality. The reported metrics include N50, L50, total assembly length, contig count, average gene content, contamination assessment, and genome completeness, thereby enabling an overall evaluation of genome assembly quality and dataset composition.

Metrics	Mean
Genome length (bp)	5,338,657
N50 (bp)	4,887,930
Number of contigs	386
GC content (%)	50.71
Number of genes	4954
Completeness (%)	99.89
Contamination (%)	0.53
Most frequent Sequence Type (ST)	ST131
Most frequent serotype	O25:H4
N (isolates included)	145
N (isolates after contamination analysis)	142

### The pangenome and its association with the resistome

**Figure 1.**
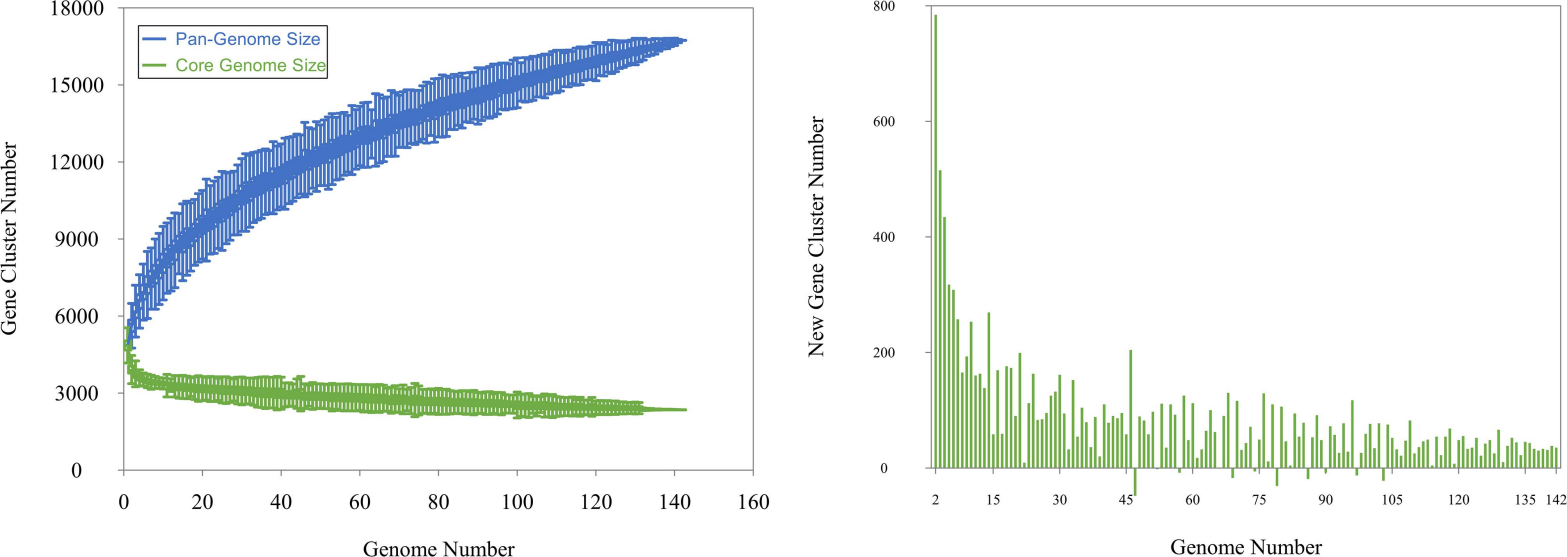
Results of pangenome. (a) Gene accumulation curve of the pangenome (blue) and core genome (green); the value represented in the box shows an open pangenome. (b) Represents the number of new genes related to the increase in *E. coli* genome size.

Analysis of the *E*. *coli* pangenome revealed a total of 16,732 genes, categorized into a persistent genome of 3,385 genes, a shell genome of 3,412, and a cloud genome of 9,935 genes, indicating an open pangenome structure (Figure 1). Pyseer identified 3,971 significant ARG – accessory gene associations involving 29 ARGs. After excluding associations involving hypothetical proteins, 3,712 associations remained. The 29 ARGs that exhibited significant associations yielded several notable findings. Among them, *aac [3]-IIe* showed the highest number of associations (*n* = 438), despite being present in only 38% of the isolates. In contrast, *blaEC-19* was involved in 125 associations but was detected in 81% (115/142) of the isolates. This pattern highlights differences in the distribution of ARGs across the population, whereby some genes participate in numerous associations despite occurring in a relatively small proportion of isolates, whereas others are more widely distributed but involved in fewer associations. Similarly, *aph(3”)-Ia*, *aac [3]-IVa*, and *blaCTX-M-27* each exhibited more than 150 associations; however, these genes were detected in less than 7% of the isolates, indicating a limited prevalence within the study population. After *blaEC-19*, the ARGs *aac(6”)-Ib-D181Y*, *sul1*, *aadA5*, *blaCTX-M-15*, *mphA*, *mrxA*, *dfrA17*, *blaOXA-1*, and *tetA* showed the highest association frequencies, with their respective associations occurring in more than 48% of the isolates (Supplementary Table 4).

**Figure 2.**
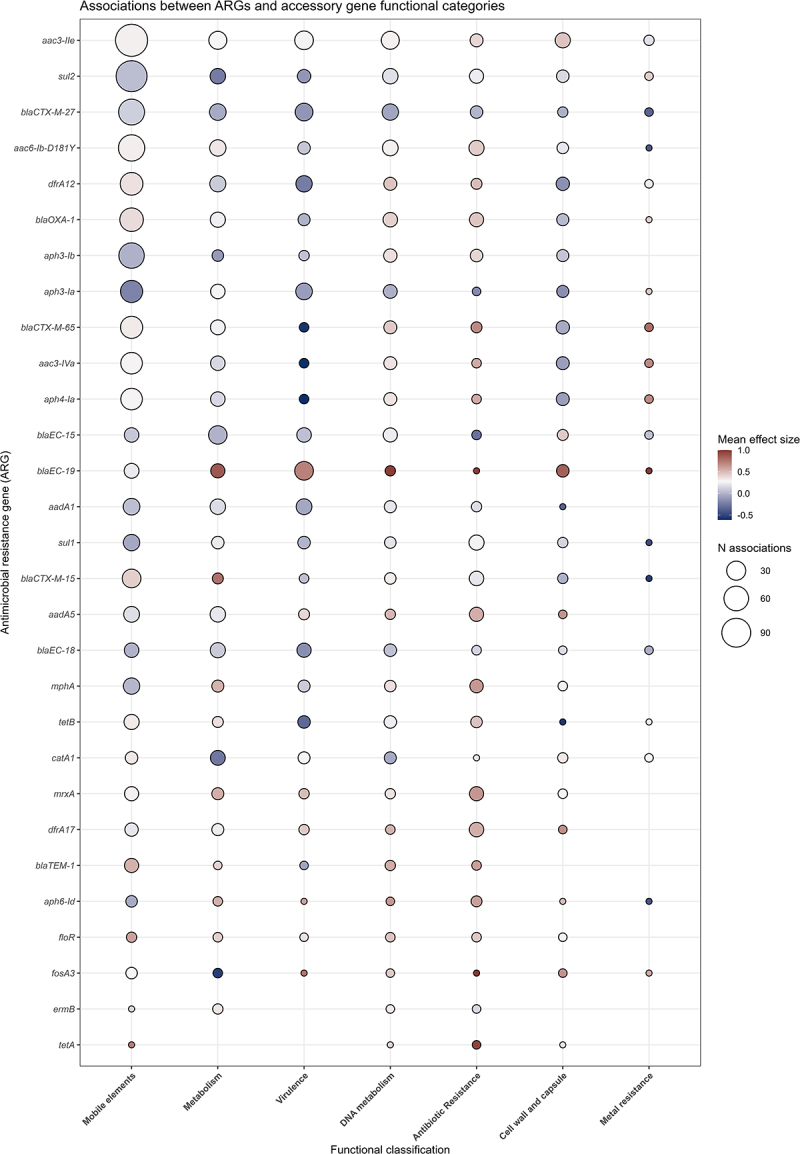
Bubble plot depicting significant associations between antimicrobial resistance genes (ARGs) and functional categories in *Escherichia coli*. ARGs are displayed on the y-axis and functional categories on the x-axis. Bubble size corresponds to the number of significant ARG–function associations, whereas colour indicates the estimated effect size. Associations were identified using Pyseer linear mixed models (LMMs), accounting for population structure through a kinship matrix derived from core-genome relatedness and sequence type (ST) covariates.

The distribution of significant associations across functional categories revealed several noteworthy patterns. Among the most prominent findings, *bla*_EC-19_ exhibited positive associations with genes involved in metabolism, virulence, DNA metabolism, antimicrobial resistance, cell wall and capsule biosynthesis, and metal resistance. In contrast, most ARGs displayed negative associations with virulence-related genes, metabolic functions, and mobile genetic elements, with the notable exceptions of *bla*_EC-19_, *fosA2*, and *aph [6]-Id*, which showed positive associations with virulence determinants. Positive associations with other antimicrobial resistance genes were primarily observed for *tetA*, *fosA3*, and *bla*_CTX-M-65_. Regarding mobile genetic elements, although numerous associations were identified within this functional category, only a limited number of ARGs, including *tetA*, *floR*, and *bla*_TEM-1_, showed positive effect sizes. Conversely, *aph(3”)-Ia*, *aph(3”’)-Ib*, *sul2*, and *bla*_CTX-M-27_ were predominantly characterized by negative associations. The remaining ARGs exhibited effect sizes close to zero, indicating no consistent positive or negative association with mobile genetic elements (Figure 2).

The *aac [3]-IIe* gene, an aminoglycoside resistance determinant, was predominantly associated with the *pap* operon that encodes P fimbriae implicated in UPEC adhesion. Similarly, *bla*_*CTX-M-15*_, a key ESBL gene, co-occurrence with *the* resistance determinants *catB3*, *aac6*, as well as with *mobQ*, DNA resolvase and Tn3-like element Tn5403 family transposase with positive effect size. These elements are commonly associated with bacterial genetics mobile elements, contributing to horizontal gene transfer. The *bla*_*EC-15*_ gene was correlated with the type II toxin-antitoxin *ChpS* and *ChpB* genes, type VI secretion system *TssE, TssK, TssL, VasI* and type 1 fimbria with positive effect size. Furthermore, *bla*_*EC-15*_ was linked to the *casABCDE* cluster, indicating potential interactions with CRISPR-Cas immunity systems. Co-occurrence of *aadA5, Sul1, bla-CTX-M-15* and *dfrA17 genes* revealed a profile of consisting of *Mrx(*A), macrolide 2”-phosphotransferase, erythromycin resistance repressor protein and *Mph(*A) family macrolide 2”-phosphotransferase, which exhibited similar association profiles with the accessory genes. Functionally, these genes are associated with macrolide, b-lactams and sulfonamide resistance mechanisms, including efflux pumps, resistance regulation, and enzymatic modification of antibiotics (Supplementary Table 2).

A comprehensive and high-resolution analysis of the *E*. *coli* pangenome revealed a highly intricate and dynamic genomic architecture characterized by the interplay between conserved core genes and a vast array of accessory genes. This structural complexity underscores the genomic plasticity of these organisms. A robust and interconnected network was discovered using a targeted analytical framework focused on the distribution and associations of ARGs, VFs and MGEs within the accessory genome.

Among the resistance determinants and MGEs, IS26, an IS family member, emerged as one of the most prominent and widely distributed insertion sequences across isolates. This element was closely linked to multiple ARGs, including *bla*_CTX-M-1_, *bla*_OXA-1_, *cat*, and *aacA4*, as well as virulence-associated loci belonging to the *pap* operon. Notably, IS26 co-occurred with Tn2, a transposase belonging to the Tn3 family, forming a highly connected network component enriched in ARGs, VFs and MEGs. These elements formed one of the most densely interconnected components of the accessory genome network.

Beyond resistance-associated regions, network analysis revealed additional accessory gene modules displaying coherent functional organization. A highly connected cluster included the lysine decarboxylase system genes *cad*A and *cad*B, the sialic acid transport-associated gene *nan*C, and the dipeptide/tripeptide permease gene *dtp*C. These loci are involved in nutrient acquisition, acid stress tolerance, and host-associated metabolic adaptation. Furthermore, genes belonging to the *pap* fimbrial operon formed an interconnected component linked to adhesion-related functions, highlighting the coexistence of resistance, virulence, and adaptive metabolic traits within the accessory genome.

**Figure 3.**
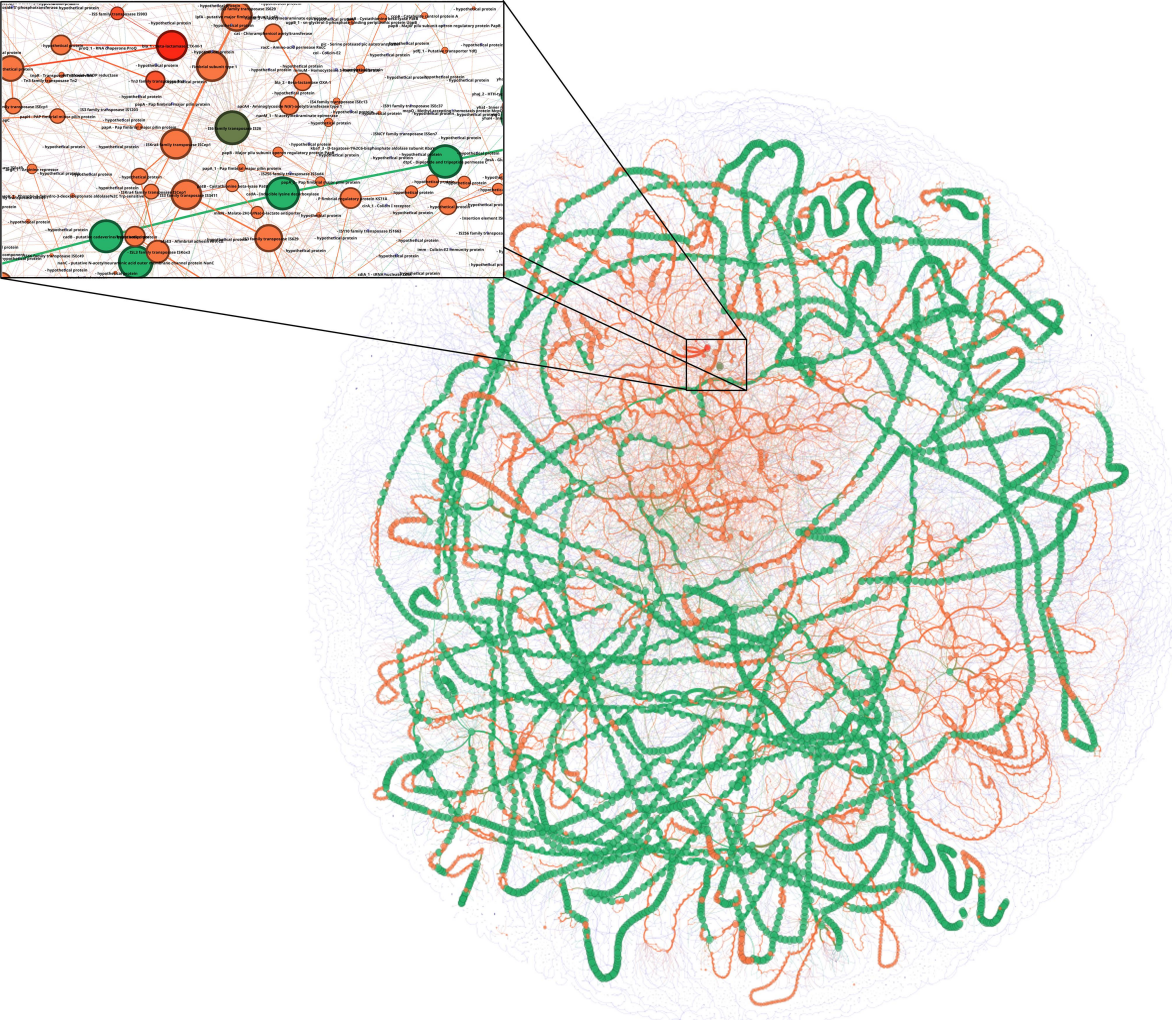
Pangenome network of *E. coli* isolates showing the distribution and relationships of gene clusters across the analyzed genomes. Nodes represent gene clusters, and edges indicate shared occurrence profiles among isolates. Persistent/core genes are shown in green, while accessory genes are shown in orange, according to the gene presence–absence matrix generated during pangenome analysis. The highlighted region corresponds to gene clusters associated with ARGs and MGEs. This region indicates where resistance-related and mobility-associated genes are concentrated within the accessory pangenome, supporting the interpretation that ARGs may be linked to horizontally acquired or variable genomic elements.

In addition to the highly connected resistance-associated elements, the accessory genome contained a diverse repertoire of insertion sequences and transposases, including *IISCep1*, *IS1203*, *IS903*, *ISKox3*, *ISEc29*, *IS629*, *IS110*, *IS1663*, *IS256*, *ISSod4*, *ISSen7*, and *ISEc27*. The widespread distribution of these elements across the accessory genome highlights the substantial mobilome diversity present among the analyzed isolates (Figure 3).

### Examination of virulome

The virulome analysis revealed distinct profiles, divided into 23 main profiles out of a total of 47 (Supplementary Table 5), with three profiles encompassing 52 isolates (36.62%). The EC1 profile comprised 22 isolates (15.49%) and was characterized by the presence of siderophore genes together with the toxin gene *sat* and adhesion-associated genes. The EC2 profile included 20 isolates (14.08%) and was distinguished by the predominance of toxin genes (*cnf1*, *hlyABCD*, and *sat*), while also harboring siderophore and adhesion-associated genes. The EC3 profile consisted of 10 isolates (7.04%) and was characterized by the presence of adhesion genes, siderophore genes, and secretion system-associated genes. Notably, all isolates belonging to these three predominant profiles were assigned to sequence type (ST) 131 (Supplementary Figure 1).

It is essential to highlight that most profiles contained genes encoding the type II secretion system (T2SS), observed in 92 isolates (65.49%) across 25 profiles. Additionally, genes associated with the type V secretion system (T5SS) were identified in nine virulence profiles, encompassing a total of 63 isolates. The T5SS was predominantly represented in profiles EC1, EC2, and EC3, mainly due to the presence of the tsh gene, which contributed substantially to the distribution of this secretion system among the isolates. Additionally, 23 profiles (EC24–EC47) displayed unique profiles compared with the rest (EC1–EC21). Profiles revealed that the main toxin gene among the E. coli isolates was sat (17 profiles), followed by east1 (11 profiles). Regarding adhesion genes, the fimABCDEFGHI operon was present in 34 profiles, followed by papABCDEFGHIJKX. Among the siderophores, the enterobactin gene was nearly ubiquitous (44 profiles), followed by yersiniabactin and aerobactin (34–32 profiles respectively) (Table 2, Supplementary Table 5).

**Table 2.** Distribution of virulence profiles among the 142 *E. coli* isolates. A total of 47 virulence profiles were identified based on the presence or absence of virulence-associated genes and operons related to toxin production, adhesion, iron acquisition, and secretion systems. Each profile represents a unique combination of virulence determinants, and the last column indicates the number of isolates assigned to each profile. The other profiles with one isolate per profile are available in Supplementary table 5.

Profile	Toxins					Adhesion					Siderophores			T2SS	T5SS		Isolates count
	*east1*	*cnf1*	*hly*ABCD	*sat*	*vat*	*fim*ABCDEFGHI	*pap*ABCDEFGHIJKX	*dra*ABCEDP	*foc*AICDFGH	*sfa*BCDGXY	*yer*	*aer*	*ent*	*gsp*	*pic*	*tsh*	
EC1	–	–	–	+	–	+	+	–	–	–	+	+	+	+	–	+	22
EC2	–	+	+	+	–	+	+	–	–	–	+	+	+	–	–	+	20
EC3	–	–	–	+	–	+	–	+	–	–	+	+	+	+	–	+	10
EC4	–	–	–	–	–	+	–	–	–	–	–	+	+	+	–	–	9
EC5	–	–	–	–	–	+	–	–	–	–	–	–	+	+	–	–	9
EC6	–	–	–	–	–	+	–	–	–	–	+	–	+	+	–	–	6
EC7	–	–	–	–	–	–	–	–	–	–	+	+	+	+	–	–	4
EC8	–	–	–	–	–	+	–	–	–	–	+	+	+	+	–	–	4
EC9	–	–	–	–	–	+	–	–	–	–	–	–	+	–	–	–	3
EC10	–	–	–	+	+	+	–	–	–	–	+	+	+	+	–	–	3
EC11	+	–	–	–	–	–	–	+	–	–	+	–	+	+	–	–	3
EC12	+	–	–	–	–	+	–	–	–	–	–	–	+	+	–	–	3
EC13	–	–	–	–	–	+	–	–	–	–	+	+	+	–	–	–	2
EC14	–	–	–	+	–	+	–	–	–	–	+	+	+	–	–	+	2
EC15	–	–	–	–	+	+	–	–	–	–	+	+	+	+	–	–	2
EC16	+	–	–	+	–	+	+	–	–	–	+	+	+	+	–	+	2
EC17	–	–	–	+	–	+	–	+	–	–	+	+	+	–	–	–	2
EC18	+	–	–	–	–	–	–	–	–	–	–	+	–	–	–	–	2
EC19	–	–	–	+	–	+	+	–	–	–	+	+	+	+	–	–	2
EC20	+	–	–	–	–	+	–	–	–	–	+	+	+	+	–	–	2
EC21	+	–	–	–	+	+	–	–	–	–	+	+	+	+	+	–	2
EC22	–	–	+	+	–	+	+	–	–	–	+	+	+	–	–	–	2
EC23	–	–	–	–	–	+	–	–	–	–	+	–	+	–	–	–	2
Others	+/-	+/-	+/-	+/-	+/-	+/-	+/-	+/-	+/-	+/-	+/-	+/-	+/-	+/-	+/-	+/-	24

### Features of phylogenomic analysis

Phylogenomic analysis of the core genome revealed a diverse organization of UPEC isolates. This diversity reflects the complex evolutionary processes that shape the pathogenicity and antibiotic resistance of these strains. The core genome comprised both homogeneous and heterogeneous clusters of isolates. Homogeneous clusters were dominated by ST131/O25:H4, characterized by a relatively stable resistome, whereas heterogeneous clusters encompassed a broader diversity of sequence types and serotypes while retaining overlapping resistance profiles. Pathotype analysis identified DAEC and EAEC strains within these clusters, with EAEC being particularly prevalent among the heterogeneous groups.

The urinary *E*. *coli* isolates also displayed distinct profiles of resistance gene distribution. In homogeneous clusters, specific resistance determinants predominated, including *aac(6’)-Ib-D181Y, aac [3]-IIe*, and *aadA5* for aminoglycosides; *bla*_*CTX-M-15*_, *bla*_*OXA-1*_, and *bla*_*EC-5*_ for beta-lactams; *dfrA17* for trimethoprim; *mph(A)* for macrolides; *sul1* and *sul2* for sulfonamides; and *tet(A)* for tetracyclines. In contrast, the heterogeneous clusters exhibited a wider variety of alleles. Notably, isolates belonging to ST44/-:H4 shared a resistance profile strikingly similar to that of the ST131/O25:H4 clade, with common genes such as *aac(6’)-Ib-D181Y, aadA5, bla*_CTX-M-1_*5, bla*_OXA-1_, *dfrA17, mph(A)*, and *sul1*, but differed in the presence of genes such as *tet(B)* and *blaEC*.

**Figure 4.**
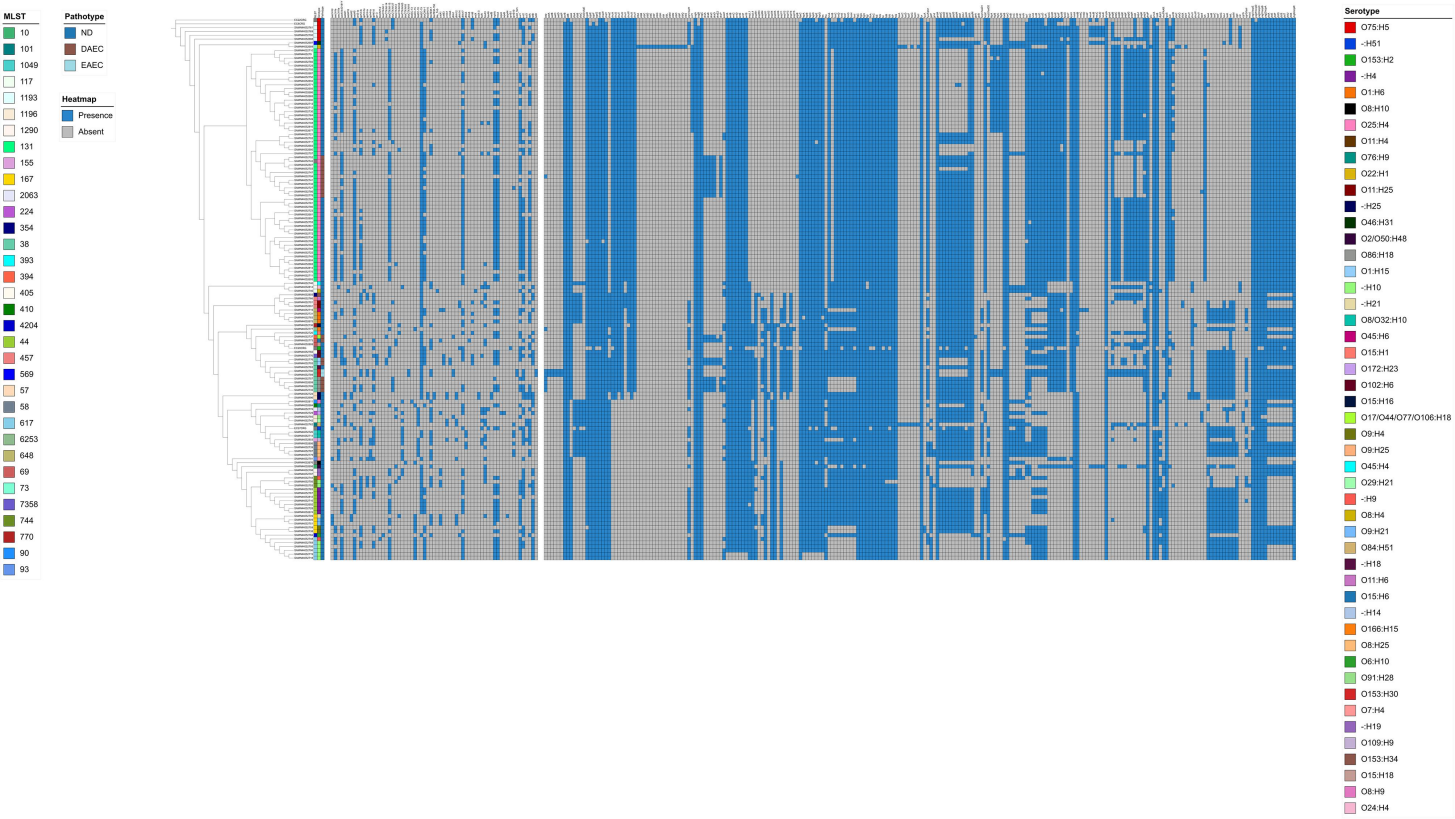
Phylogenomic tree based on the core-genome alignment of the analyzed isolates, with accompanying heatmaps indicating the presence or absence of antimicrobial resistance genes (ARGs) and virulence factors (VFs). ARGs and VFs were identified using abricate, and gene presence was defined as hits with ≥80% sequence identity and gene coverage. Blue filled cells represent detected genes, whereas empty cells represent genes not detected under these criteria. Metadata columns indicate multilocus sequence type (MLST), pathotype, and serotype for each isolate.

Analysis of the virulome in UPEC revealed a wide array of ARGs shared among isolates. Notably, a specific group of DAEC isolates responsible for urinary tract infections possesses a distinct set of genes (*afaA*, *afaB-I*, *afaC-I*, and *afaD*) that facilitate bacterial adhesion. These isolates were ST131 and O25:H4 and shared the same VFs as the UPEC isolates located in the same clade, with identical ST and serotype. Generally, the clade predominantly occupied by ST131 and serotype O25:H4 exhibited a largely homogeneous distribution of ARGs. In contrast, a highly heterogeneous distribution was observed in the clade with a wide variety of STs and serotypes. Additionally, within this heterogeneous clade of STs, a distinct pathotype, EAEC, was identified, consisting of two isolates with unique genes (*aggA*, *aggB*, *aggC*, and *aggD*) that encode a special fimbria in enteroaggregative *E*. *coli* (Figure 4).

### Summary of genomic characteristics of E.Coli isolates

The genomic composition of the E. coli collection is summarized in Table 3. Among the 142 isolates analyzed, ST131 was the most prevalent sequence type, comprising 60 isolates (42.3%). The resistome was dominated by *blaEC-19*, detected in 115 isolates (81.0%), followed by *dfrA17* and *mphA*, each identified in 88 isolates (62.0%). Several virulence-associated genes, including *allB, csgA, csgC, flgC, flgG, fliE, fliG, fur, gndA*, and *ibeC*, were conserved across the collection, being present in all 142 isolates (Table 3).

**Table 3.** Key genomic characteristics of the *Escherichia coli* isolate collection. Summary of the top 10 results from 142 *E. coli* isolates, including sequence type (ST) distribution, the most prevalent antimicrobial resistance genes (ARGs), and the most frequent virulence-associated genes (VFs). Values are presented as number of positive isolates and percentage of the total collection.

Category	Feature	Count	Frequency (%)
Sequence Type	131	60	42,3
	38	9	6,3
	44	7	4,9
	617	6	4,2
	1193	5	3,5
	167	5	3,5
	58	4	2,8
	648	4	2,8
	117	3	2,1
	Other STs	34	23,9
Antimicrobial Resistance Genes	*bla*_EC-19_	115	81,0
	*dfrA17*	88	62,0
	*mphA*	88	62,0
	*sul1*	87	61,3
	*mrxA*	85	59,9
	*aadA5*	81	57,0
	*bla*_CTX-M-15_	81	57,0
	*aac6-Ib-D181Y*	74	52,1
	*tetA*	74	52,1
	*bla*_OXA-1_	69	48,6
Virulence Factors	*allB*	142	100,0
	*csgA*	142	100,0
	*csgC*	142	100,0
	*flgC*	142	100,0
	*flgG*	142	100,0
	*fliE*	142	100,0
	*fliG*	142	100,0
	*fur*	142	100,0
	*gndA*	142	100,0
	*ibeC*	142	100,0

## Discussion

### Composition of UPEC clones in Ecuador

UPEC is the predominant etiological agent of UTIs worldwide, affecting hundreds of millions of people annually. Regional genomic surveillance of these strains is of great public health relevance [37]. This study provides the first genomic characterization of UPEC isolates from Ecuador, offering crucial insights into the local epidemiology of this clinically significant pathogen. By applying comparative genomics, we elucidated the pangenome, virulome, and resistome structures of our UPEC isolates. This genomic framework significantly improves our understanding of the evolution and dissemination of UPEC virulence and resistance in Ecuador, providing a basis for future surveillance strategies, therapeutic decision-making, and infection control tailored to the regional context.

The genetic variability identified by our analysis in UPEC within Ecuador encompasses 142 genomes derived from clinical isolates, which showed a conserved core of 3,385 genes, alongside a significant number of accessory genes (9,935 cloud genes), suggesting an important genetic plasticity. This genetic plasticity likely corresponds to an evolutionary response to differential antimicrobial pressures present in the environment, consistent with the results of multiple investigations on the virulence and resistance strategies employed by UPEC, as is the case with the ST131 clone [38], which was the predominant lineage in the sequences used for the phylogeny analysis, as well as shown in other Latin American countries [19]. The most striking finding was the marked predominance of the ST131, predominantly of the O25:H4 serotype, aligning with its global status as a cosmopolitan, pandemic, and multidrug-resistant clone, and underscoring its role as a primary driver of UTIs in Ecuador. The ST131 lineage has been reported to be subdivided into three main clades (A, B, and C), with a 79 % prevalence of clade C [39]. In our analyses, the sequences belonging to the ST131 lineage likely belonged mainly to the pandemic clade C, according to the resistome and virulome profiles. We found that VFs characteristics of UPEC strains, such as *fyuA* and *chuA* [40], may be employed in diagnostic efforts aimed at targeting. ST131 fitness is closely linked to the *fimH30* variant of type 1 fimbriae and delayed *fim* operon expression caused by *fimB* insertion [41]. This genetic refinement contributes to the successful colonization and persistence of our population. The analysis confirms a dynamic functional structure, a hallmark of UPEC, capable of facilitating rapid adaptation to environmental pressures.

In addition to the prevalence of ST131 in Ecuador, the in-depth genomic characterization of our four recent genomic submissions from pediatric clinical isolates also identified clinically significant lineages, highlighting a more complex local epidemiology with a multi-resistance profile associated with high-risk strains. We determined that three of our isolates (EC22CRG, EC27CRG, and EC38CRG) were part of the ST58 lineage, a high-risk clone associated with an increased risk of sepsis progression [42]. Despite belonging to the same lineage, these three isolates belonged to different serotypes (O75:H5, O15:H6, and O6:H10), with different virulence and resistance capacities, thus showing genomic flexibility within this clone. The fourth isolate (EC8CRG) belonged to the ST1193 lineage, which is also a high-risk UPEC pathogen owing to its fluoroquinolone resistance and ability to cause UTIs and systemic infections [43]. The coexistence of these high-risk clones (ST58 and ST1193), distinct from ST131, reveals a multifaceted epidemiological threat posed by UPEC strains circulating in Ecuador. However, our analysis also revealed that local epidemiology with other high-risk clones is more complex.

### Virulome structure and its implications for pathogenic risk

We performed a comprehensive virulome analysis to assess the pathogenic potential of these clones. VFs associated with adhesion processes and siderophores are fundamental in infectious processes, particularly for *E*. *coli*, as adhesion genes facilitate infection and colonization [44]. In contrast, siderophores contribute to iron acquisition for survival in the host. Our virulomes were organized into twenty-three distinct groups, with three main profiles (EC1, EC2, and EC3) accounting for a significantly large proportion (36.61 %). This organization highlights the varied, yet structured, VFs supporting UPEC pathogenicity. Adhesion genes (*fim* and *pap*) and siderophores (*aer* and *ent*) were the most prevalent. The near-ubiquity of the *fim* operon (found in 34 profiles) and the high prevalence of the pap operon, along with siderophore systems such as enterobactin (44 profiles), yersiniabactin, and aerobactin (34, and 32 profiles respectively), are consistent with an essential role in adherence and iron acquisition in the establishment and maintenance of UTIs. The involvement of *fim* has been implicated in activating TLR4 in patients with cystitis and pyelonephritis [45]. Moreover, genes such as *cnf1* and *hylABCD* were found to be less abundant but occasionally present. Profile EC2 was particularly notable, composed exclusively of ST131 isolates and characterized by this potent combination of toxin genes (*cnf1*, *hlyABCD*, *sat*). This combination is associated with increased tissue damage, cytotoxicity, and immune evasion, identifying the EC2 subpopulation as a potentially high-risk subset within Ecuador’s already successful ST131 lineage.

### ARGs-MGEs dynamics

In parallel with the diversity of VFs, we characterized the resistome of the isolates, focusing on their association with MGEs. The *E*. *coli* resistome is significantly influenced by ARGs linked to MGEs, such as plasmids, which facilitate horizontal gene transfer and contribute to the dissemination of antimicrobial resistance in UPEC and other bacterial pathogens [46,47]. Consistent with this, 29 identified ARGs showed relevant co-occurrence with accessory genes, highlighting the important role of the accessory genome in shaping resistance profiles.

Among the resistance-associated modules, IS26 emerged as a prominent network component, co-occurring with set of ARGs (*bla*_CTX-M-1_, *bla*_OXA-_*1*, *catB3*, and *aac6)* and virulence loci (pap operon). In addition, IS26 was associated with Tn2, forming a highly connected module enriched in ARGs and MGEs. Similar IS26-mediated resistance structures have been widely reported in animal reservoirs and clinical UTI isolates and are considered important contributors to ARG dissemination [48–50]. Although the fragmented assemblies prevent confirmation of physical linkage, the recurrent co-occurrence of these elements suggests that they frequently occupy similar genomic contexts. Beyond resistance determinants, the accessory genome also contained interconnected modules composed of genes associated with metabolic adaptation and host colonization, including *cadA*, *cadB*, *nanC*, and *dtpC*. Together with the pap operon, these findings indicate that accessory genome diversification in UPEC involves not only antimicrobial resistance but also traits related to fitness and host adaptation.

Within ST131, the core genomes of clade C were relatively conserved, whereas the accessory genome had relevant differences in ARG content and associated MEGs in this lineage [51]. In our dataset, some ST131 groups exhibited stable resistance profiles, while others carried a broader repertoire of ARGs and accessory genes despite belonging to the same lineage. This variability highlights the ongoing diversification of ST131 through changes in accessory genome content while maintaining a conserved genomic backbone [52]. This pattern reinforces the role of the accessory genome as a major driver of microevolution and diversification within this successful lineage.

### Co-ocurrence patterns between virulence and resistance determinats

We provide compelling genomic evidence of a strong synergy between VFs and ARGs, which is facilitated by MGEs. The association of *aac [3]-IIe* with the pap operon and *blaEC* with the *paaABCDEFGH* metabolic operon suggests that ARG-MGEs or VFs-MGEs genetic modules are not independent but are often physically linked and transferred in a parallel manner, a phenomenon with increasing reports in bacterial clones of public health relevance [53]. This co-occurrence implies that selective pressure derived from antibiotic use can directly increase VFs diversity and vice versa, as the co-occurrence of ARGs and VFs associated with MGEs is an inflection point for the evolution of the so-called superbacteria [54]. For example, aminoglycoside use could not only select for *aac [3]-IIe* but also promote the retention and spread of the adjacent pap operon, leading to the circulation of more resistant and virulent strains, a condition observed in successful UPEC outbreaks worldwide [55]. Furthermore, the relationship between certain ARGs suggests a potential adaptive advantage beyond resistance, possibly facilitating nutrient acquisition and persistence in the host environment, as some resistance and virulence mechanisms have been linked to improved metabolic capacity [56].

### UPEC pangenome and its adaptability in the region

Analysis of the UPEC pangenome in Ecuador reveals an open structure with a substantial accessory component, providing an essential genomic framework for understanding the dynamic distribution of UPEC VFs in Ecuador. The organization of the virulome into 23 profiles, with three main profiles (EC1, EC2, and EC3) that group 36.61 % of the isolates, demonstrates that the variability of VFs is not random but is structured in specific combinations associated with successful lineages. These three main profiles refer to the number of isolates corresponding to them but not necessarily the number of VFs. For example, EC2 had the highest number of VFs [9], in addition to their abundance of isolates, and showed great concern in the region. EC1, EC2 and EC3 possess the Secreted Autotransporter Toxin (*sat*) toxin, which is referenced with the ability to cause cellular damage in *E*. *coli* infections [57], providing the ability to evade the immune system [58], and also as a toxin of interest in the probiotic strain *E*. *coli* Nissle 1917 (ECN) [59]. The presence of the complete set for the production of the fim1 adhesin gene [60], and the complete *pap* operon that gives them the necessary machinery to produce one of the most important VFs in the UPEC infectious process [61], projecting them as highly virulent pathogenic strains. Regarding siderophores, the presence of the *ent* operon was observed in the sequences of the analyzed UPEC strains, which encodes enterobactin, a crucial VF that allows the acquisition of iron from the host, exacerbating urinary tract infections [62]. The presence of *iucA*, *iucB*, *iucC*, and *iucD* VFs was also observed in all profiles except EC18, which encodes the aerobactin system, in the UPEC pangenome, allowing them to evade the immune defense of lipocalin 2, aggravating UTIs [63]. Other profiles, such as EC16, and EC21, each with more than eight detected VFs, presented two isolates each, suggesting limited epidemiological relevance in the context of UPEC in Ecuador. Regarding the predominance of ST131, which has also been widely reported among UPEC isolates [64], this sequence type was identified in multiple virulence profiles, including EC1, EC2, and EC3. However, EC2 was distinguished by a marked enrichment of siderophore and toxin genes, whereas EC1 and EC3 harbored comparatively fewer toxin-associated determinants. These findings suggest that the plasticity of the accessory genome enables the diversification and specialization of subpopulations within the same high-risk lineage, generating distinct virulence repertoires despite a shared clonal background. This specialization is evidenced by the near ubiquity of adhesion systems, such as the *fim* operon, as reported by other authors [65], and iron acquisition systems, such as enterobactin, which constitute a “solid core” of virulence. In contrast, accessory elements, such as salmochelin or yersiniabactin systems, confer particular adaptive advantages. Thus, the pangenome architecture facilitates the modular evolution of virulence, where a core set of essential VFs is complemented by an accessory reservoir that can be recombined to optimize pathogenic fitness in response to specific selective pressures, as observed for the specialization of pathotypes such as DEC and UPEC [66].

In a parallel and mechanistically interconnected manner, the strong association observed between ARGs, and the accessory genome underlines the fundamental role of HGT in resistome evolution. The HGT phenomenon is common in hospital environments [67] and is enhanced by MGEs [68]. The finding that 29 identified ARGs significantly co-occurred with genes from the accessory pangenome indicates that MGEs would mediate their acquisition and dispersal. Studies in *E*. *coli* suggest that the combination of genes in pangenomes is not random but follows deterministic rules and is possibly predictable [69]. However, environmental interactions should not be excluded [70]. This phenomenon is exemplified by the widespread distribution of the commonly referenced IS26 insertion element in bacteria [71], which is linked to a critical set of ARGs (*bla*_CTX-M-1_, *bla*_OXA-1_, *aacA4*) and the *pap* virulence operon. Increased expression of *bla*_CTX-M-1_ has been reported in patients with sepsis [72] and its existence in cockroaches [73], highlighting its vectorial importance in the dispersal of ARGs. The identification of a wide range of other accessory insertion sequences (IS) reinforces the notion of a complex and active mobilome, consistent with studies in *E*. *coli* [74–76]. Beyond mere physical co-occurrence, identified functional associations, such as the link between the aminoglycoside resistance gene *aac [3]-IIe* and the P-fimbria *pap* operon or between the beta-lactamase *blaEC* and the phenylacetic acid degradation operon *paaABCDEFGH*, would suggest a co-selection process. Recent studies have shown *that aac [3]-IIe* is among the most abundant ARGs for Enterobacterales, with aminoglycoside resistance being a point of attention for health [77], in addition to the association of this and beta-lactamase with MGEs [8].

### Regional implications

The UPEC pangenome characterized in Ecuador demonstrates this complexity, with direct and profound regional implications. The statistical association of ARGs with accessory content, especially with MGEs, outlines a genomic landscape that favors HGTs as the protagonist of the aggravation of this situation. This genomic organization suggests that local antimicrobial pressures in Ecuador not only select resistant clones, but also actively drive the assembly and dissemination of complex genetic modules that co-occurrence resistance and virulence determinants. Thus, the Ecuadorian UPEC pangenome constitutes a global reservoir of adaptation, where the accessory component predisposes the accelerated evolution of high-risk strains through, for example, the involvement of MGEs. This dynamic poses a significant challenge, particularly regarding hypervirulent and multidrug-resistant phenotypes, which should be the primary focus of regional surveillance and control strategies.

### Limitations

This study has several limitations. First, our analysis relied on publicly available genomes, and for many isolates, the metadata, clinical details, epidemiological context, collection dates, and geographic origin were incomplete or missing entirely. Second, access to phenotypic susceptibility testing data was not available; for that reason, the AMR findings reflect genomic resistance potential only and should not be taken as confirmation of actual clinical resistance. Third, the genome assemblies were highly fragmented (median: 538 contigs per genome), which made it difficult to determine physical linkage, identify plasmid context, or confirm whether resistance genes (ARGs), mobile genetic elements (MGEs), and virulence factors truly co-occur on the same genetic element. Finally, our association analyses may be skewed by the underlying population structure most notably the heavy over-representation of the ST131/O25:H4 lineage in the dataset. For all these reasons, the associations we report are best understood as genome-level co-occurrence profiles. Confirming them will require plasmid-resolved assemblies, long-read sequencing, or direct experimental work.

## Conclusions

This study is the first to report the global genomic analysis of UPEC in Ecuador, revealing a convergence of high-risk clones with marked antimicrobial resistance and a diverse virulome, all facilitated by an open pangenome architecture. The predominance of certain lineages, coupled with the identification of cosmopolitan clones, underscores the multifaceted epidemiological threat. Specifically, the strong association between ARGs and the accessory genome, mediated by an active mobilome, highlights HGT as a key factor in adaptation to local selective pressures. This knowledge compels a paradigm shift in regional surveillance strategies, moving beyond clone tracking and toward the utilization of cutting-edge technology. Indeed, this genomic knowledge is fundamental for guiding empirical therapy, laying the groundwork for control policies, and mitigating the public health crisis posed by multidrug-resistant UTIs in Ecuador.

## Supplementary Material

Supplementary table 5.xlsx

Supplementary Table 1.xlsx

Suplementary Table 3.xlsx

Supplementary Table 4.xlsx

Supplementary Figure 1.png

Supplementary Table 2.xlsx

## Data Availability

All sequences used in this study are available at NCBi under bioprojects PRJNA1168579 and PRJNA1293794. The following supplementary material can be downloaded from: 10.6084/m9.figshare.30700817.

## Abbreviations

AMR: Antimicrobial Resistance
*bla*KPC: β-lactamase *Klebsiella pneumoniae* carbapenemase gene
KPC: *Klebsiella pneumoniae* Carbapenemase
ESKAPE: *Enterococcus faecium, Staphylococcus aureus, Klebsiella pneumoniae, Acinetobacter baumannii, Pseudomonas aeruginosa, Enterobacter spp*
ESBL: Extended-Spectrum *β-Lactamase*
SRA: Sequence Read Archive
NCBI: National Center for Biotechnology Information
bp: base pairs
TSD: Target Site Duplication
ST: Sequence Type
MLST: Multilocus Sequence Typing
NTE: Non-Tn4401 Element
IS: Insertion Sequence
Tn: Transposon
